# Suppression of miR-204 enables oral squamous cell carcinomas to promote cancer stemness, EMT traits, and lymph node metastasis

**DOI:** 10.18632/oncotarget.7745

**Published:** 2016-02-26

**Authors:** Cheng-Chia Yu, Pei-Ni Chen, Chih-Yu Peng, Chuan-Hang Yu, Ming-Yung Chou

**Affiliations:** ^1^ Institute of Oral Sciences, Chung Shan Medical University, Taichung, Taiwan; ^2^ School of Dentistry, Chung Shan Medical University, Taichung, Taiwan; ^3^ Department of Dentistry, Chung Shan Medical University Hospital, Taichung, Taiwan; ^4^ Oral Medicine Research Center, Chung Shan Medical University, Taichung, Taiwan; ^5^ Institute of Biochemistry, Microbiology and Immunology, Chung Shan Medical University, Taichung, Taiwan

**Keywords:** oral squamous cell carcinomas, miR-204, stemness, EMT, metastasis

## Abstract

The feature of oral squamous cell carcinomas (OSCC) is commonly metastasizing to locoreginal lymph nodes, and the involvement of lymph nodes metastasis represents the one of important prognostic factors of poor clinical outcome. MicroRNAs (miRNAs) have been shown to be key players of cancer-related hallmarks including cancer stemness, EMT (epithelial-mesenchymal transition), and metastaisis. Herein we showed that OSCC-derived ALDH1^+^ cancer stem cells (OSCC-CSCs) express lower level of miR-204, and miR-204 over-expression suppresses cancer stemness and *in vivo* tumor-growth of OSCC-CSCs. miR-204 binds on their 3′UTR-regions of Slug and Sox4 and suppressing their expression in OSCC-CSCs. On the contrary, down-regulation of miR-204 significantly increased cancer stemness and the lymph nodes incidence of orthotopic animal models. Furthermore, co-knockdown with sh-Slug and sh-Sox4 synergistically rescued miR-204-supressing cancer stemness and EMT properties. Clinical results further revealed that a miR-204^low^Slug^high^Sox4^high^ signature predicted the worse survival prognosis of OSCC patients by Kaplan-Meier survival analyses. Up-regulated miR-204-targeting Slug and Sox4 by epigallocatechin-3-gallate (EGCG) treatment significantly inhibited the proliferation rate, self-renewal capacity, and the percentage of ALDH1^+^ and CD44^+^ cells in OSCC-CSCs Oral-feeding of EGCG effectively alleviated tumor-progression in OSCC-CSCs-xenotransplanted immunocompromised mice through miR-204 activation. In conclusion, miR-204-mediated suppression of cancer stemness and EMT properties could be partially augmented by the anti-CSCs effect of EGCG.

## INTRODUCTION

Oral squamous cell carcinomas (OSCC) is the sixth most common cancer type worldwide with poor prognosis [1]. Unfortunately, treatments including extensive surgery, radiotherapy, chemotherapy or concurrent chemo/radiotherapy are not effective for patients with advanced OSCC due to tumor recurrence, metastasis, and poor response to chemotherapy and radiotherapy [1]. The cervical lymph node metastasis is the major cause of death for OSCC patients [1]. Thus it is of great significance to identify molecular mechanisms and effective therapies for OSCC lymph node metastasis. Cumulative evidence suggest that cancer stem cells (CSCs) have been considered as the main cause of metastasis, resistance to chemotherapy and radiotherapy in OSCC [2–4]. Recent reports suggested that CD44 [5], CD133 [6], aldehyde dehydrogenase (ALDH) [7], membrane GRP78 [8], side population [9] and c-Met [10] could be the markers to identify the CSCs from OSCC. Our previous studies have demonstrated that OSCC-CSCs not only present elevated epithelial-mesenchymal transition (EMT) markers but also are highly metastatic, tumorigenic, and resistant to radiotherapy and chemotherapy [3]. Thus, attempt to develop strategies targeting CSCs as the key for successful treatment against OSCC.

MicroRNAs (miRNAs), a class of small noncoding RNAs regulating the gene expression by binding to the 3′ untranslated region (UTR) of target mRNAs, has been involved in many important biological processes by controlling gene expression at the post-transcriptional level [11–13]. Several studies have also shown that miR-204 is frequently downregulated in glioma [14], breast cancer [15], gastric cancer [16], colorectal cancer [17], papillary thyroid carcinoma. [18], and non-small-cell lung carcinoma [19], suggesting tumor suppressive role of miR-204 in human tumorigenesis. miR-204 has been linked to regulate the cancer stemness. For example, miR-204 attenuates sphere-forming ability, stemness markers (SOX2, NANOG, KLF4, OCT4, and CD133) expression, invasiveness, and in vivo growth in glioma [20]. However, the roles of miR-204 as well as its downstream targets in the regulation of cancer stemness in OSCC remain unclear.

In this report, our studies illustrated an novel-regulatory role of the miR-204-targeting Sox4 and Slug co-expression in the regulation of cancer stemness, EMT, and lymph node metastasis of OSCC cells. Through the upregulation of miR-204 by EGCG appears to suppress cancer stemness in OSCC-CSCs.

## RESULTS

### miR-204 overexpression targets CSCs properties in vitro and in vivo

**Figure 1:**
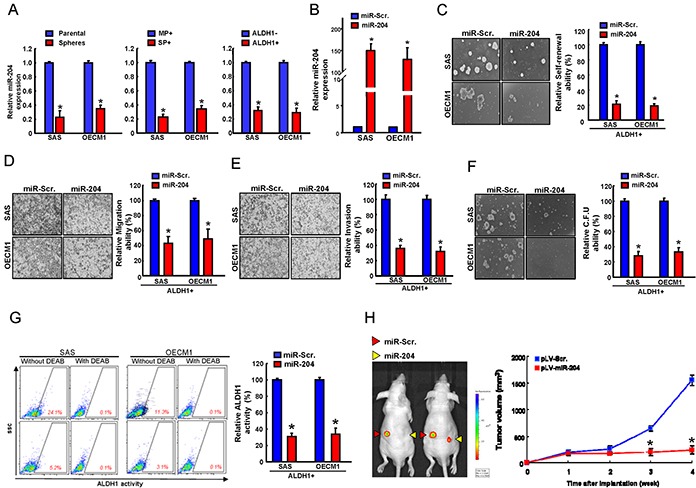
miR-204 suppresses cancer stemness in vitro and in vivo **A.** miRNA levels of miR-204 in sphere-forming and parental cells (left), major population and side population cells (middle), and ALDH^+^ and ALDH1^−^ cells (right) were assessed by miRNA quantitative real-time PCR and are presented as relative fold-changes. **B.** The efficiency of miR-204 overexpression in OSCC-CSCs by quantitative real-time PCR and presented as relative fold-changes. **C.** Representative images (left) and quantification (right) of spheres formation in OSCC-ALDH1+ cells transfected with the indicated vectors. The formation of spheres was monitored for up to 14 days, and the rate of sphere formation was calculated. pLV-miR-204- and pLV-ctrl-transfected OSCC-CSCs cells were analyzed for migration **D.** invasion **E.** and colony-formation ability **F. G.** ALDH1 activity of pLV-miR-204- and pLV-ctrl-transfected OSCC-CSCs cells were assigned for the flow cytometry analysis. **H.** Nude mice were subcutaneously injected with pLV-miR-204 and pLV-ctrl transfected OSCC-CSCs cells in various amounts as indicated, and the mice were monitored for4 weeks. Results are means ± SD. *, p < 0.05 vs. Control.

Previously, we have showed that miR-204 was downregulated in ALDH1+CD44+ OSCC-CSCs by miRNAs microarray analysis [3]. To further investigate whether miR-204 plays a role in the identity of OSCC-CSCs, the quantitative RT-PCR analysis were performed to confirm that miR-204 levels were low in ALDH1^+^, SP^+^, and sphere-forming OSCC cells but high in ALDH1^−^, MP, and parental cells (Figure 1A). To demonstrate the significance of miR-204 in regulating the cancer stemness in ALDH1^+^ OSCC-CSCs, miR-204 was overexpressed in OSCC-CSCs by lentiviral-based delivery system (pLV/miR-204). A scrambled vector-transfected control (pLV/miR-Scr.) was also generated simultaneously. The effect of ectopic miR-204 over-expression in OSCC-CSCs was validated by quantitative miRNA RT-PCR analysis (Figure 1B). The results of functional analysis showed that overexpression of miR-204 significantly suppressed sphere formation ability (Figure 1C) and migration/invasion capacity (Figure 1D & Figure 1E) in OSCC-CSCs, as compared with their control cells. miR-204 overexpression not only decreased the colony formation ability (Figure 1F) but also percentages of ALDH1^+^ cells (Figure 1G). Animal study demonstrated that overexpression of miR-204 effectively inhibited tumor-initiating property of OSCC-CSCs in nude mice model (Figure 1H). Collectively, our results suggest that miR-204 repress the cancer stemness properties of OSCC cells in vitro and in vivo.

### miR-204 directly targets the 3′UTR of SOX4 and SLUG

**Figure 2:**
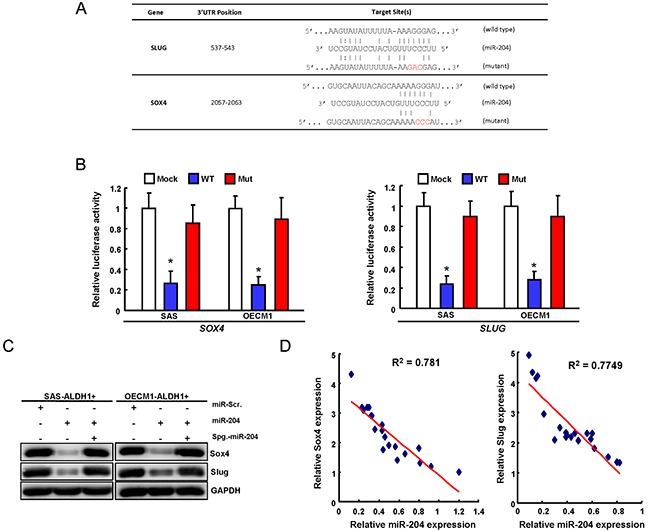
miR-204 targets Sox4 and Slug in OSCC-CSCs **A.** Schematic presentation of the constructed Slug and Sox4 3′UTR reporter plasmids were used in this study. **B.** The wild-type and mutated (Mut) Sox4 and Slug reporter plasmids were co-transfected withmiR-204 **o**r empty vector. The luciferase activity of each combination was assessed and was presented with wild-type (WT) and mutated (Mut) reporter plasmids. The results of the luciferase assays indicated that only WT reporter activity was inhibited by miR-204. **C.** The protein expression levels of Slug and Sox4 OSCC-CSCs transfected indicated plasmids were analyzed by western blot. **D.** Correlations between miR-204/ Sox4 (left panel) and miR-204/ Slug (right panel) in OSCC patients tissues was analyzed.

With using the Target Scan software, we identified potential miR-204 targeting sites in the 3′UTR regions of SOX4 and SLUG. To pinpoint the miR-204 target sequences in the 3′UTRs of SOX4 and SLUG, reporter plasmids which contained either full-length or mutated forms of the 3′UTR region of SOX4 and SLUG were constructed (Figure 2A). Luciferase reporter assays demonstrated that miR-204 reduced the luciferase activity of reporter plasmids containing full-length SOX4 and SLUG 3′UTR (Figure 2B). However, when the potential SOX4 and SLUG targeting site was mutated, miR-204 no longer inhibited the luciferase activity (Figure 2B). The protein levels of SOX4 and SLUG were decreased in the miR-204 -overexpressing OSCC-CSCs (Figure 2C). The results showed that the protein reduction level in of SOX4 and SLUG was abolished when co-transfected with miR-204 sponge (Figure 2C). Clinical results revealed that SOX4 and Slug expression was inversely correlated with miR-204 in the tissues of OSCC patients (Figure 2D). Taken together, our data indicate that miR-204 can directly bind the 3′UTRs of SOX4 and Slug and negatively correlate their expression in OSCC patients.

### Correlation of the miR-204^low^Sox4^high^Slug^high^ expression signature with poor overall survival of OSCC patients

**Figure 3:**
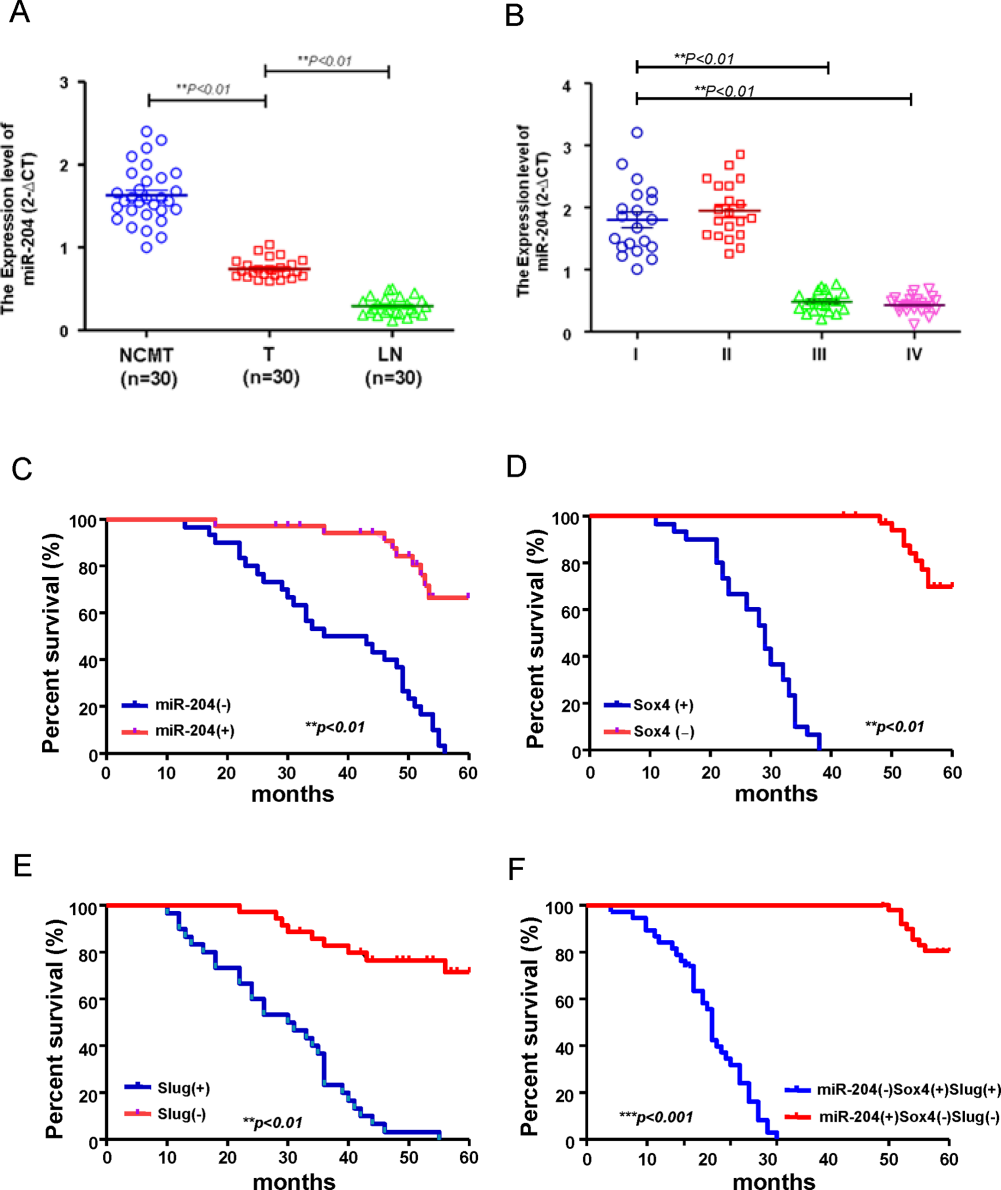
The miR-204^low^Sox4^high^Slug^high^ signature predicts poor survival in OSCC patients **A.** Adjacent adjacent noncancerous matched tissues (NCMT; n=30), and paired tissue samples from tumor (T; n=30) as well as lymph node metastatic (LN; n=30) lesions in OSCC patients were subjected to analysis for the expression levels of miR-204. **B.** Samples from patients with different stages (stage I to IV) of OSCC were collected and subjected to miRNAs qPCR analysis for miR-204 level. Kaplan–Meier analysis of overall survival of OSCC patients was stratified according to the Sox4 score **C.** the Slug score **D.** the miR-204 score **E.** grouped as miR-204(+)Sox4(−)Slug (−) or miR-204 (−)Sox4 (+)Slug (+) **F.**

To further assess whether the relative expression levels of miR-204 may be involved in OSCC patients, we examined miR-204 levels in adjacent noncancerous matched tissues (NCMT) samples, local tumor (T) samples and metastatic lymph nodes (LN) samples from OSCC patients using miRNA real-time RT-PCR analysis. As shown in Figure 3A, the relative expression level of miR-204 was significantly lower in local tumor (T) and lymph nodes metastasis (LN) samples compared to the adjacent noncancerous matched tissues (NCMT) tissues (Figure 3A). A negative correlation between low miR-204 expression levels and advanced stage was revealed by further analysis of miR-204 expression levels in different stages of OSCC specimens (Figure 3B). Kaplan–Meier survival analysis revealed that patients with negative expression of miR-204 had poorer overall survival than those with positive expression of miR-204 (Figure 3C). OSCC patients with negative expression of Sox4 (Figure 3D) or Slug (Figure 3E) had better overall survival compared with those with positive expression of Slug or Sox4. Most of importance, patients with an expression profile of miR204^low^Slug^high^Sox4^high^ had the lowest survival rate compared with those with miR-204^high^Slug^low^Sox4^low^ (Figure 3F). These findings indicate that miR204^low^Slug^high^Sox4^high^ as a signature of OSCC patients progression.

### miR-204 depletion promotes cancer stemness and enhances lymph node metastasis

**Figure 4:**
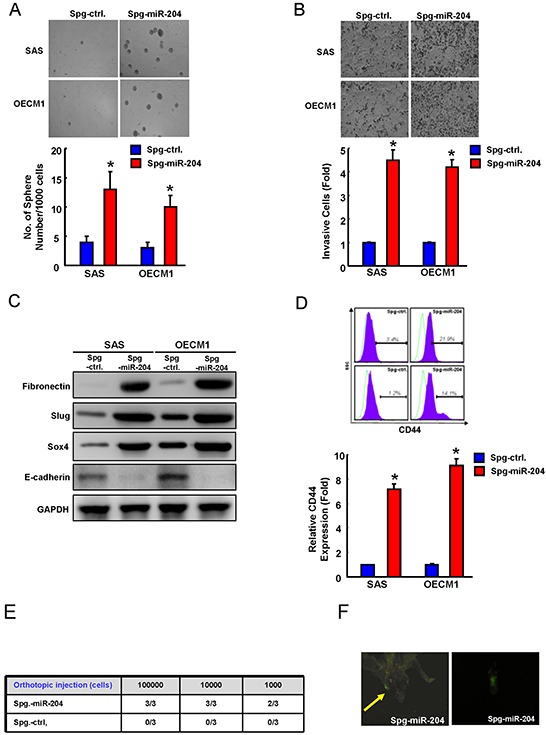
Suppression of miR-204 is able to enhance cancer stemness and metastasis Spg-miR-204- and Spg-ctrl-transfected ALDH1^−^ cells were assigned for the spheres formation assay **A.** invasion assay **B.** EMT traits **C.** and CD44 positivity **D. E.** Orthotopic injection of various amounts Spg-miR-204 or Spg-ctrl-transfected ALDH1^−^ OSCC cells (from 1000 to 100,000 cells) into nude mice, and the mice were monitored for 6 weeks lymph node metastasis development (right, n=3). **F.** Representative image of lymph node metastasis development in orthotopic Spg-miR-204- ALDH1^−^ cells-transplanted mice. Results are means ± SD. *, p < 0.05 vs. Control.

We further examined whether the decrease miR-204 expression correlated with the cancer stemness and metastasis. By using an miRNA-SPONGE strategy, we knocked down miR-204 in ALDH1^−^ cells isolated from SAS and OECM1 cells, and subjected these cells to functional and molecular analysis. The sphere formation ability (Figure 4A) and the invasion cells (Figure 4B) was all elevated upon miR-204 knockdown (Spg-miR-204) as compared to the control (Spg-ctrl) cells. miR-204 down-regulation elevated mesenchymal markers including N-cadherin and Vimentin and repressed epithelial marker (E-cadherin) (Figure 4C). Prince et al has identified CD44 also as functional marker of OSCC-CSCs [5]. miR-204 down-regulation also increased CD44 positivity in OSCC cells (Figure 4D). We orthotopically injected 10^3^, 10^4^ or 10^5^ miR-204-knockdown OSCC cells into immunodeficient mice and measured lymph node metastasis incidence. The incidence of lymph node metastasis *in vivo* in orthotopic mice was increased by knocking down of miR-204 in OSCC cells (Figure 4E & Figure 4F).

### Sox4 and Slug co-expression dominates miR-204-mediated cancer stemness and EMT

**Figure 5:**
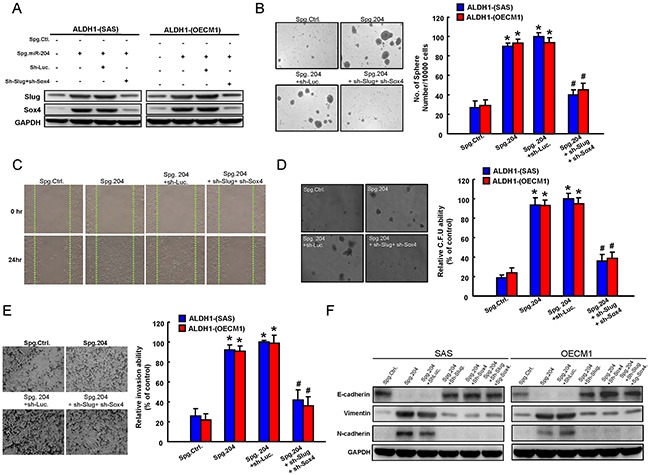
Involvement of Slug and Sox4 in miR-204-regulated cancer stemness and EMT **A.** ALDH1^−^ OSCC cells transfected with Spg-miR-204, sh-Sox4, or sh-Slug, as indicated, were analyzed by Western blot for the levels of cellular Slug and Sox4. ALDH1^−^ cells were subjected to sphere formation **B.** wound-healing assay **C.** colony-forming assay **D.** and invasion assay **E.** that were transfected with indicated plasmids. **F.** ALDH^−^ OSCC cells infected with Spg-miR-204, Spg-miR-204+Sh-Luc, Spg-miR-204+Sh-Sox4, Spg-miR-204+Sh-Slug, or Spg-miR-204+Sh-Sox4+Sh-Slug were analyzed by western blot for the expression level of the indicated EMT markers. Results are means ± SD. *, p < 0.05 vs. Control.

The functional involvement of Sox4 and Slug in miR-204-mediated cancer stemness and EMT was further clarified. Initially, co-knockdown of Sox4 and Slug expression in Spg-miR-204 OSCC cells was verified by western blotting (Figure 5A). Silencing of endogenous miR-204 induced spheres-forming capability in ALDH1^−^ cells, which would be blocked by co-knockdown of Sox4 and Slug (Figure 5B). The wound-healing (Figure 5C), invasion abilities (Figure 5D), and clonogenicity (Figure 5E) in ALDH1^−^-OSCC cells were increased in Spg-miR-204 OSCC cells. Furthermore, co-silencing of Sox4 and Slug in Spg-miR204-treated ALDH1^−^ cells partially counteracted these phenomenons (Figure 5C–5E). With western blotting, we demonstrated that Spg-miR204 induced a pattern of up-regulated mesenchymal-like proteins (N-cadherin and Vimentin) and down-regulated epithelial protein (E-cadherin) in ALDH1^−^ cells, were reversed by Sox4 and/or Slug down-regulation (Figure 5F).

### EGCG treatment impaired cancer stemness and in vivo tumor growth through miR-204 activation

**Figure 6:**
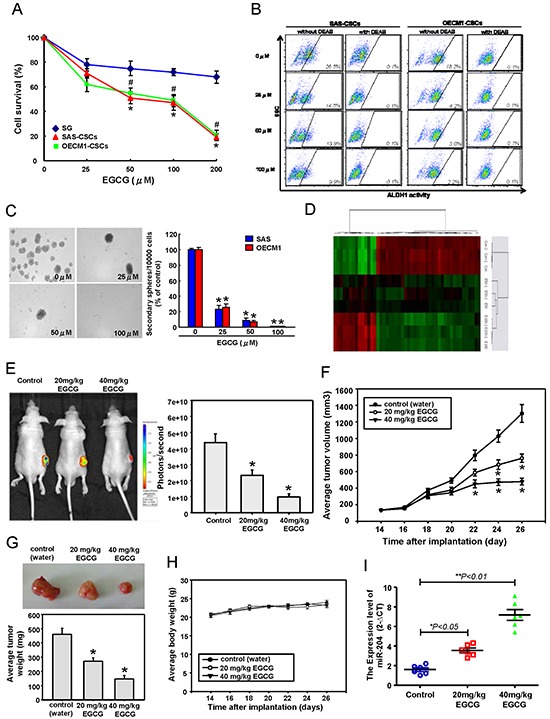
Anti-CSCs effects of EGCG in OSCC through miR-204 induction **A.** Cell survival of SG and OSCC-CSCs treated with various concentrations of EGCG up to 200 μM was assessed by MTT assay. OSCC-CSCs treated with or without EGCG were subjected to **B.** ALDH1 activity and **C.** self-renewal secondary sphere-forming assay. **D.** miRNA microarray and bioinformatic analyses were conducted to compare the miRNAs expression patterns between the indicated cells. After subcutaneous implantation of OSCC-CSCs, BALB/c nude mice (*N* = 6 for each group) were oral-feeding treated with water or EGCG and then photographed and analyzed for the bioluminescence signal **E.** tumor volume **F.** average tumor weight **G.** and average mice body weight **H. I.** Mice were sacrificed, and tumor sections as indicated treatments were assessed for relative miR-204 expression.

Accumulated evidence has suggested that dietary compounds target cancer stemness, and therefore offer a promising approach for cancer prevention and therapy [21]. Moreover, epigallocatechin-3-gallate (EGCG) has been shown to suppress the cancer stemness and tumor initiation ability of breast [22] and nasopharyngeal cancer cells [23]. In lung cancer cells, EGCG-regulated miRNAs have been shown to be involved in the epigenetic regulation of oncogenicity [24]. We examined the effect of EGCG on normal oral epithelial cells (SG) and OSCC-CSCs isolated from SAS and OECM1 cells. EGCG inhibited the proliferation rate of OSCC-CSCs in a dose-dependent manner, whereas the inhibition on SG cells proliferation was limited (Figure 6A). These data demonstrated that EGCG was specific and acted almost exclusive on CSCs, rather than normal, non-transformed cells. We then evaluate the potential role of EGCG in modulating the CSCs properties of OSCC cells, and found that EGCG decreased the percentage of ALDH1^+^ cells (Figure 6B & Supplementary Figure S1A), self-renewal capacity (Figure 6C), the invasiveness (Supplementary Figure S1B) of OSCC-CSCs. Control and EGCG-treated OSCC-CSCs were subjected to miRNAs microarray analyses to attempt to identify the EGCG-modulated specific miRNAs that mediate cancer stemness of OSCC-CSCs (Figure 6D). miRNA RT-PCR analysis showed that miR-204 expression was significantly increased in OSCC-CSCs with EGCG dose-dependent treatment (Supplementary Figure S1C). Accordantly, EGCG -treated OSCC-CSCs also decreased the levels of Sox4 and Slug, which our data implicated as targets of miR-204 (Supplementary Figure S1D & Figure 1E). To verify the in anti-tumor effects of EGCG against OSCC-CSCs *in vivo*, immunocompromised mice bearing OSCC-CSCs xenografts were treated with water or EGCG by oral gavage. Notably, tumor formation in all recipients was reduced following xenotransplantation of OSCC-CSCs that received oral gavage EGCG treatment on day 26 as compared to control animals (Figure 6E). Moreover, by day 26, EGCG feeding dose-dependently induced a reduction in tumor volume (Figure 6F) and tumor weight (Figure 6G) and without any apparent signs of toxicity as evidenced by body weight monitoring (Figure 6H). miRNA real-time RT-PCR analysis of the sections of these tumors showed that EGCG-treated tumor had increased miR-204 expression in comparison to those from control OSCC-CSCs tumors (Figure 6I). A schematic representation of the EGCG-targeting Sox4 and Slug in the regulation of the cancer stemness and EMT resulting lymph node metastasis of OSCC cells is shown (Supplementary Figure S1F).

## DISCUSSION

In recent years, miRNAs have recently been linked to regulate the properties of cancer stem cells (CSCs) [25, 26]. For example, miR-1 repressed cancer stemness, proliferation, and migration properties in breast cancer cells through targeting Wnt/β-catenin pathway [27]. Our previous studies have analyzed miRNA profiles of OSCC-CSCs and non-CSCs by miRNA microarray and identified down-regulation of miR-204 in OSCC-CSCs [3]. However, the functional roles of miR-204 in OSCC-CSCs remain understudied. Here, we identified a significant down-regulation of miR-204 in OSCC-CSCs (Figure 1). Subsequently, functional studies using lentiviral-based miR-204 overexpression suppressed the CSCs properties of OSCC-CSCs through targeting3′UTR regions of Slug and Sox4 (Figure 1 & Figure 2). Clinical results suggested that low miR-204 expression in OSCC patients correlated with a poor patient survival rate and advanced stage tissues (Figure 3). Knockdown of miR-204 in OSCC cells enhanced cancer stemness and metastasis, while co-knockdown of Slug and Sox4 effectively reversed these phenomena (Figure 5). To our knowledge, this is the first report demonstrating miR-204 expression is crucial for maintaining the cancer stemness and metastasis of OSCC.

Epithelial-mesenchymal transdifferentiation (EMT) program, a critical process involved in the transdifferentiation of polarized epithelial cells into an invasive mesenchymal phenotype, has emerged as a central driver of tumor malignancy including tumorigenicity, metastasis, and cancer stemness [28]. Sox4, the member of the Sox (Sry-related high-mobility group box) family of transcription factors, has been functions as a master mediator in EMT [29]. Researchers have shown that Sox4 could promote EMT properties, the number of CD44^high^/CD24^low^ population, and sphere-forming ability in breast cancer cells [30]. Sox4 was also demonstrated to involve in TGF-β-induced EMT properties through EZH2 modulation [31]. Slug, the member of the Snail superfamily, has been demonstrated that functions as a transcriptional repressor by specifically binding to E-box motif [32, 33]. Several experimental and clinical studies have revealed that Slug has been implicated also in pathological alterations of the phenotype associated with the acquisition of invasiveness and cancer stemness by tumors [34, 35]. In the study, we demonstrated that Spg-miR-204 induced a pattern of up-regulated mesenchymal-like proteins (N-cadherin and Vimentin) and down-regulated epithelial protein (E-cadherin) in ALDH1^−^ OSCC cells, which were reversed by Slug or/and Sox4 down-regulation (Figure 5F). Future research delineates the details of how miR-204 regulates its Slug/Sox4 axis, and how these interactions influence the EMT and cancer properties of OSCC remains to be determined. Further research effort is needed in this area.

Epigallocatechin-3-gallate (EGCG), the most abundant polyphenol in green tea, which has been shown to inhibit cancer stemness properties in breast, nasopharyngeal, liver, and colon cancers [22, 23, 36]. These results showed that specifically targeting CSCs might be regarded as the key for cancer treatment. Recent reports have shown that miRNAs in the anti-tumor effects of EGCG in several types of malignant cancers. Wang et. al has demonstrated that EGCG inhibits proliferation and anchorage-independent growth of lung cancer cells via miR-210 induction [24]. The present study first showed that EGCG inhibited in vitro and in vivo tumorigenicity through the miR-204 activation, which resulted in the inhibition of the self-renewal, tumor initiation, and metastatic properties of OSCC (Figure 6). Recurrence of cancers after conventional therapeutic treatments is thought to be due to re-emergence of chemotherapy-resistant CSCs [37]. Because chemotherapeutic drugs, such as cisplatin or fluorouracil (5-FU), are already used to treat advanced OSCC clinically, development of an adjuvant therapy to target CSCs from natural products that are highly safe, and can strengthen the effect of chemotherapeutic drugs should be a feasible strategy for OSCC.

In conclusion, the present study showed that miR-204 was downregulated in lymph node metastasis tissues of OSCC patients. Low expression of miR-204 was correlated with poor overall survival of OSCC patients. miR204-mediated inhibition of Sox4 and Slug consequently disrupted the maintenance of the cancer stemness and EMT properties of OSCC in vitro and in vivo. Elevating miR-204 expression by EGCG treatment appears to be a promising therapeutic modality to target OSCC-CSCs.

## MATERIALS AND METHODS

### Cell culture and reagents

The Smulow–Glickman (S-G) human gingival epithelial cell line was original from F.H. Kasten, East Tennessee State University, Quillen College of Medicine, Johnson City, TN [38]; SAS, a high-grade tumorigenic human tongue squamous cell carcinoma, was obtained from the Japanese Collection of Research Bioresources (Tokyo, Japan) [39]; Human gingival squamous carcinoma cells (OECM-1) were provided from Dr. C. L. Meng (National Defense Medical College, Taipei, Taiwan) grown in RPMI supplemented with 10% FBS. Cells were cultured at 37°C containing 5% CO2. EGCG was purchased from Sigma Chemical Co. (St. Louis, MO) and was dissolved in DMSO (Merck, Darmstadt, Germany) as a stock solution of 100 mM. Just before use, EGCG was further diluted in culture medium to appropriate final concentrations.

### Isolation of ALDH1+ OSCC-CSCs

ALDH+ cells were stained with ALDEFLUOR assay kit (StemCell Technologies, Inc., Vancouver, BC, Canada) following our previous report and were sorted by FACSAria II cell sorter (BD Biosciences, San Jose, CA, USA) [3].

### qRT–PCR analysis for miR-204 analysis

miRNAs qRT–PCR was performed using TaqMan miRNA assays with specific primer sets (Applied Biosystems, Carlsbad, Calif) for miR-204 levels detection. All reagents and protocols were from Applied Biosystems, and detection was performed using a 7900HT fast real-time PCR system [40].

### Tumorsphere-forming assay

Tumor cells were dissociated and cultured as tumorspheres in modified DMEM/F-12 supplemented with N2 (R&D), 10 ng/mL epidermal growth factor (EGF, Invitrogen), 10 ng/mL basic fibroblast growth factor (bFGF, Invitrogen), and penicillin/streptomycin at 103 live cells/low-attachment six-well plate (Corning Inc., Corning, NY), and the medium was changed every other day until the tumor sphere formation was observed in about 2 weeks [3].

### Migration/invasion assay

Cell migration and invasion assay was assayed using 24-well plate Transwell® system with a polycarbonate filter membrane of 8-μm pore size (Corning, United Kingdom) assay kit as previously described [8].

### CD44 staining by flow cytometry analysis

Cells were stained with anti-CD44 antibody conjugated to phycoerythrin (Miltenyi Biotech., Auburn, CA, USA), with labeling according to the manufacturer's instructions. Red (>650 nm) fluorescence emission from 10,000 cells illuminated with blue (488 nm) excitation light was measured with a FACSCalibur (Becton Dickinson) using CellQuest software. In cell-sorting experiments, cells were labeled and sorted using FACSAria (BD Biosciences) [21].

### Cell proliferation/survival determination by MTT assay

1×10^4^ cells/well in 0.1 % DMSO or different concentration of silibinin-containing medium and cultured at 37°C for 24hr. Cell proliferation/survival was determined by MTT (3-(4,5-dimethylthiazol-2-yl)-2,5-diphenyl tetrazolium bromide) assay for evaluation of cell proliferation,. The MTT test was conducted according to previously used protocols [21].

### Western blot

The extraction of proteins from cells and western blot analysis were performed as described. Samples (15 mL) were boiled at 95°C for 5 min and separated by 10 % SDS-PAGE. The proteins were wet-transferred to Hybond-ECL nitrocellulose paper (Amersham, Arlington Heights, IL, USA). The following primary antibodies were listed in Supplementary Table S1. Immunoreactive protein bands were detected by the ECL detection system (Amersham Biosciences Co., Piscataway, NJ, USA) [3].

### MiR-204 sponge

Oligos for miR-204 sponge, and scramble construction were constructed using a pcDNA 6.2-GW/EmGFP-miR plasmid (Invitrogen). MicroRNA SPONGE sequence design was based on previous report [41]. Further multiple copy amplifications were done with recovery of BamH1 and XhoI digested fragments and subcloned into pcDNA 6.2-GW/EmGFP-miR plasmid [3].

### Soft agar colony forming assay

The soft agar colony forming assay was conducted according to previously used protocols [3].

### OSCC tissues acquirement and preparation

The study was approved by the institutional review board of Chun Shan Medical University Hospital. Resected tissues from OSCC patients, who gave informed consent for the use of their tissue, were harvested at surgery. Pairs of tumor (T) and adjacent noncancerous matched tissues (NCMT) parts, as well as lymph node (LN) metastatic OSCC lesions were obtained from surgical procedures send to the pathology lab for frozen section diagnosis. The information regarding the different stages of OSCC patients is described in listed in Supplementary Table S2. Tumor tissues were microscopically screened to have >70% of their areas occupied by tumor cells. The remaining specimens were snap frozen in liquid nitrogen and stored at −80°C for quantitative miRNAs real-time reverse transcription–PCR (qRT-PCR) (Applied Biosystems, Foster City, CA, USA) [3].

### Imaging measurement of tumor growth in nude mice

All procedures involving animals were in accordance with the institutional animal welfare guidelines of the Chung Shan Medical University. For the nude mice xenograft model, 5-6 weeks old immuno-deficient nude mice (BALB/c nu/nu mice) weighing 18-22 g were used. The mice were housed with a regular 12 h light/12 h dark cycle and ad libitum access to standard rodent chow diet (Laboratory Rodent Diet 5001, LabDiet, St. Louis, MO) and were kept in a pathogen-free environment at the Laboratory Animal Unit. OSCC-CSCs (1×10^4^cells/0.2 mL/mouse) were injected subcutaneously into the right front axilla. 14 days postimplantation, the mice were randomly divided into three groups (N = 5 for each group) and fed by oral gavage with water (control) and EGCG (20 and 40 mg/day/kg). The day of cell implantation was designated day 0. Imaging measurement was performed using an IVIS50 animal imaging system (Xenogen Corp.). The volume was calculated (according to the following formula: [length × width^2^]/2), and then analyzed using Image-Pro Plus software. Body weight was assessed daily after cell injection. After 26 days, the animals were euthanized, and the primary tumors were weighed and for miR-204 analysis [21].

### Orthotopic xenograft tongue cancer mouse model

All the animal practices in this study has been approved and in accordance with the Institutional Animal Care and Use Committee (IACUC) of the Chung Shan Medical University. Cells from each stable Spg-Ctrl. or Spg-miR-204 cells will be injected into tongues of BALB/c nude mice (6-8 weeks). Thirty days after tumor injection, all mice will be sacrificed and necropsy will be performed with removal of tongue tumors and cervical lymph nodes. All tumors and cervical lymph nodes were fixed in formalin and embedded in paraffin for H&E staining for evaluation of metastasis [4].

### Statistical analysis

Statistical Package of Social Sciences software (version 13.0) (SPSS, Inc., Chicago, IL) was used for statistical analysis. Student's t test was used to determine statistical significance of the differences between experimental groups; p values less than 0.05 were considered statistically significant. The level of statistical significance was set at 0.05 for all tests.

## SUPPLEMENTARY MATERIALS FIGURES AND TABLES


