# Supplementary material for: Suppression of miR-204 enables oral squamous cell carcinomas to promote cancer stemness, EMT traits, and lymph node metastasis
Source: Oncotarget. 2016 Feb 26;7(15):20180–92. doi: 10.18632/oncotarget.7745 (PMC4991446; doi:10.18632/oncotarget.7745)
Supplement: Supplementary file 1 [file oncotarget-07-20180-s001.pdf]

SUPPLEMENTARY MATERIALS AND METHODS

Side population analysis

Cells were resuspended at 1×10^6/mL in pre-warmed DMEM with 2% FCS. Hoechst 33342 dye was added at a final concentration of 5µg/mL in the presence or absence of verapmil (50µM; Sigma) and was incubated at 37°C for 90 min with intermittent shaking. At the end of the incubation, the cells were washed with ice-cold HBSS with 2% FCS and centrifuged down at 4°C, and resuspended in ice-cold HBSS containing 2% FCS. Propidium iodide at a final concentration of 2 µg/mL was added to the cells to gate viable cells. The cells were filtered through a 40-µm cell strainer to obtain single cell suspension before analysis. The Hoechst 33342 dye was excited at 357 nm and its fluorescence was dual-wavelength analyzed (blue, 402–446 nm; red, 650–670 nm). Analyses were done on FACSAria (BD, San Diego, CA).

Lentiviral-mediated RNAi for silencing Sox4 or Slug

The pLV-RNAi vector, which co-expressing GFP protein in infected host cells, was purchased from Biosettia Inc. (Biosettia, San Diego, CA, USA). The method of cloning the double-stranded shRNA sequence is described in the manufacturer's protocol. Oligonucleotide sequence of lentiviral vectors expressing shRNA that targets human Bmi1 and ADAM10 were synthesized and cloned into pLVRNAi to generate a lentiviral expression vector. Sh-Sox4: 5' AAAAGCAATATGCCGTGTAGAATTTGGAT CCAAATTCTACACGGCATATTGC-3'; Sh-Slug: 5'-AA AAGGTTGCCATTGTTGAACTATTGGATCCAATAG TTCAACAATGGCAACC-3';Sh-Luc:5'-CCGGACTTA CGCTGAGTACTTCGAACTCGAGTTCGAAGTACTC AGCGTAAGTTTTTG-3' was utilized for experimental control. Lentivirus production was performed as above.

Microarray analysis and bioinformatics

Total RNA was extracted from cells using Trizol reagent (Life Technologies, Bethesda, MD, USA) and the Qiagen RNAeasy (Qiagen, Valencia, CA, USA) column for purification. Microarray analysis was performed using the Human OneArray miRNA v2 (Phalanx Biotech, Belmont, CA, USA). Fluorescence intensities were measured and scanned separately using Molecular Dynamics Axon 4100A scanner and assessed using GenePixPro software.. Data analysis was performed using GenePix Pro 3.0.5.56 (Axon Instruments, USA) and GeneSpring GX 7.3.1 software (Agilent, Palo Alto, CA). The average-linkage distance was used to assess the similarity between two groups of gene expression profiles as described below. The difference in distance between two groups of sample expression profiles to a third was assessed by comparing the corresponding average linkage distances (the mean of all pair-wise distances (linkages) between members of the two groups concerned). The error of such a comparison was estimated by combining the standard errors (the standard deviation of pair-wise linkages divided by the square root of the number of linkages) of the average-linkage distances involved. Classical multidimensional scaling (MDS) was performed using the standard function of the R program to provide a visual impression of how the various sample groups are related.

SUPPLEMENTARY FIGURE AND TABLES

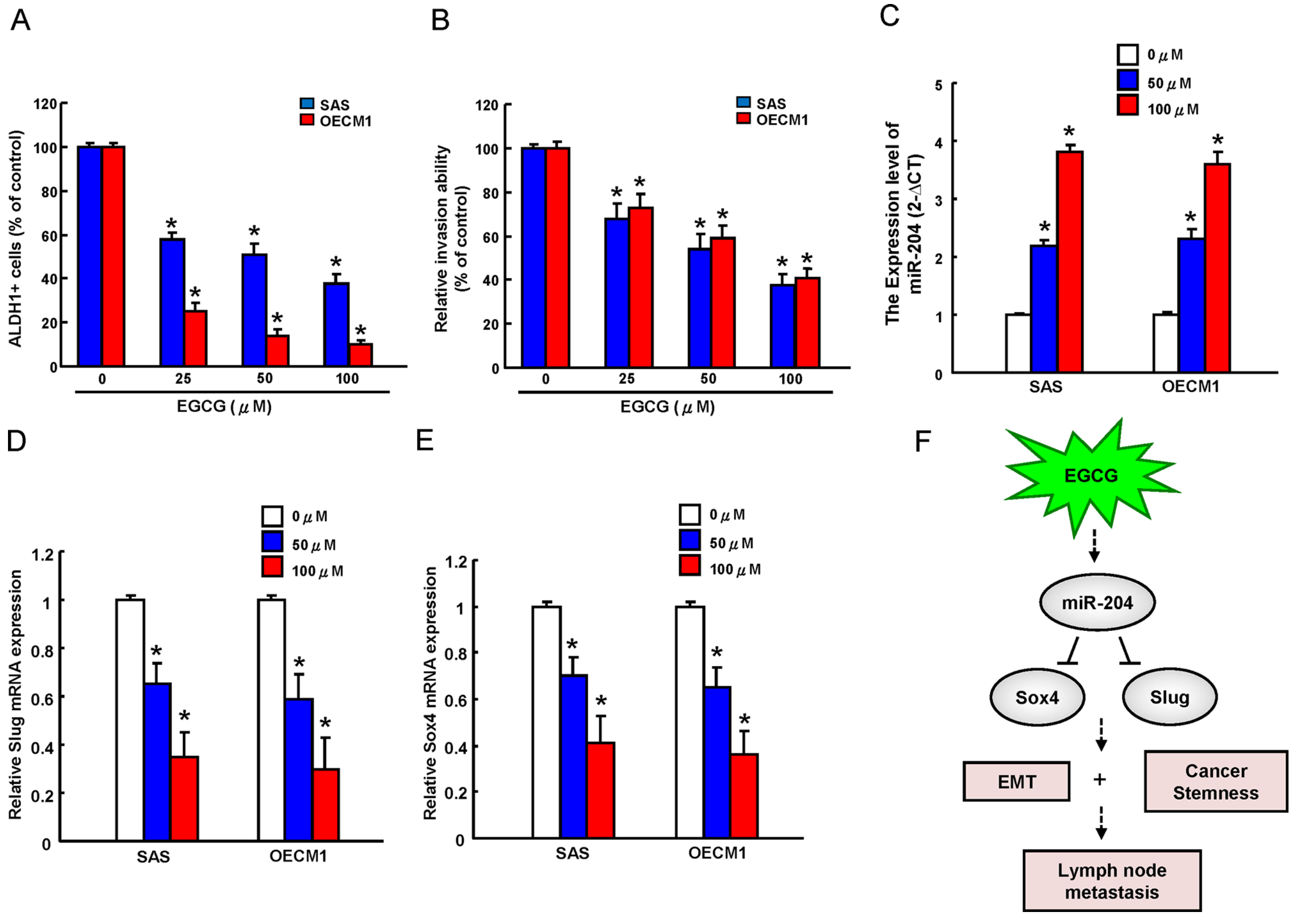

Supplementary Figure S1: Different concentration EGCG-treated OSCC-CSCs were subjected to ALDH1 activity A. and invasion assay **B. C.** miRNA qPCR analysis was applied to analyzed the relative miR-204 expression level in EGCG dose-dependently treated OSCC-CSCs. Real-time RT-PCR analysis to determine the transcript expression levels of Slug **D.** and Sox4 **E.** in OSCC-CSCs. **F.** A schematic representation of the EGCG-targeting Sox4 and Slug in the regulation of the cancer stemness and EMT resulting lymph node metastasis of OSCC cells is shown.

Supplementary Table S1: The antibodies information for western blotting

Protein	Assay	Antibody	Origin
Vimentin	WB	mmab	#66001, Santa Cruz Biotechnology, Inc.
Fibronectin	WB	rpab	#7939, Santa Cruz Biotechnology, Inc.
Sox4	WB	rpab	Sigma-Aldrich, Inc.
E-cadherin	WB	mmab	#8426, Santa Cruz Biotechnology, Inc.
GAPDH	WB	rpab	Ab9385, Abcam, Inc

Abbreviations: WB, Western blot; mmab, mouse monoclonal antibody; rpab, rabbit polyclonal antibody

Supplementary Table S2: Clinicopathological parameters of OSCC patients

Parameters	Stage I and Stage II	Stage III and IV
Age (years)	31-61	32-58
Sex (M/F)	39/1	40/0
Site		
Buccal mucosa	16	17
Tongue	23	23
Other sites	1	0
Histopathologic diagnosis		
Well differentiation cancer	23	4
Moderate differentiation cancer	13	12
Poor differentiation cancer	4	24
